# Effects of Lipid-Lowering Drugs on High-Density Lipoprotein Subclasses in Healthy Men—A Randomized Trial

**DOI:** 10.1371/journal.pone.0091565

**Published:** 2014-03-24

**Authors:** Heiner K. Berthold, Manfredi Rizzo, Nadine Spenrath, Giuseppe Montalto, Wilhelm Krone, Ioanna Gouni-Berthold

**Affiliations:** 1 Department of Internal Medicine and Geriatrics, Bielefeld Evangelical Hospital (EvKB), Bielefeld, Germany; 2 BioMedical Department of Internal Medicine and Medical Specialties, University of Palermo, Palermo, Italy; 3 Euro-Mediterranean Institute of Science and Technology, Palermo, Italy; 4 Center for Endocrinology, Diabetes and Preventive Medicine, University of Cologne, Cologne, Germany; University of Bristol, United Kingdom

## Abstract

**Context and Objective:**

Investigating the effects of lipid-lowering drugs on HDL subclasses has shown ambiguous results. This study assessed the effects of ezetimibe, simvastatin, and their combination on HDL subclass distribution.

**Design and Participants:**

A single-center randomized parallel 3-group open-label study was performed in 72 healthy men free of cardiovascular disease with a baseline LDL-cholesterol of 111±30 mg/dl (2.9±0.8 mmol/l) and a baseline HDL-cholesterol of 64±15 mg/dl (1.7±0.4 mmol/l). They were treated with ezetimibe (10 mg/day, n = 24), simvastatin (40 mg/day, n = 24) or their combination (n = 24) for 14 days. Blood was drawn before and after the treatment period. HDL subclasses were determined using polyacrylamide gel-tube electrophoresis. Multivariate regression models were used to determine the influence of treatment and covariates on changes in HDL subclass composition.

**Results:**

Baseline HDL subclasses consisted of 33±10% large, 48±6% intermediate and 19±8% small HDL. After adjusting for baseline HDL subclass distribution, body mass index, LDL-C and the ratio triglycerides/HDL-C, there was a significant increase in large HDL by about 3.9 percentage points (P<0.05) and a decrease in intermediate HDL by about 3.5 percentage points (P<0.01) in both simvastatin-containing treatment arms in comparison to ezetimibe. The parameters obtained after additional adjustment for the decrease in LDL-C indicated that about one third to one half of these effects could be explained by the extent of LDL-C-lowering.

**Conclusions:**

In healthy men, treatment with simvastatin leads to favorable effects on HDL subclass composition, which was not be observed with ezetimibe. Part of these differential effects may be due to the stronger LDL-C-lowering effects of simvastatin.

**Trial Registration:**

ClinicalTrials.gov NCT00317993

## Introduction

There is great clinical interest in raising levels of high-density lipoprotein cholesterol (HDL-C), given its epidemiologically well-established inverse association with atherosclerosis and cardiovascular disease (CVD) risk [1]. However, quantification of HDL-C, the cholesterol carried by HDL particles (HDL-P), may not fully capture HDL-related risk [1], [2]. For example, some forms of genetically low [3] or high HDL-C [4], [5] do not correspond to expected differences in coronary heart disease risk. For decades, high-density lipoproteins and HDL-C levels were considered synonymous and modulation of HDL-C levels by drug therapy held great promise for the prevention and treatment of CVD. Recent failures of drugs that raised HDL-C without reducing CVD events [6], [7] or atherosclerosis [8] have also fueled interest away from a cholesterol-centric view towards alternative indexes of HDL quantity (i.e., HDL-P or apolipoprotein A-I) or possibly HDL “quality”, such as particle size, subclass distribution [9], or various measures of HDL functionality [2], [10], [11]. Recently it was shown for example that HDL particle concentrations were associated with intima-media thickness and incident coronary events independently of atherogenic lipoproteins and HDL-C [12]. In the setting of potent statin therapy, HDL particle number may be a better marker of residual risk than HDL-C or apoA-I [13].

Statins have been shown to modestly increase or not alter HDL-C concentrations. Moreover, while they have a moderate effect on LDL subclass distribution [14], [15], their influence on HDL composition remains controversial and is overall not well investigated [16].

Ezetimibe, a cholesterol absorption inhibitor, is able to reduce low-density lipoprotein cholesterol (LDL-C) by 15–25% when given as monotherapy or added to ongoing statin treatment [17]. Due to the complementary mechanisms of action of ezetimibe and statins (inhibition of cholesterol absorption and synthesis, respectively) and to their additive effects on LDL-C-lowering, their combination is widely used to achieve reductions in LDL-C of up to 60% [18].

The HDL family represents a highly heterogeneous group of plasma lipoproteins, ranging in density between d = 1.063–1.21 g/ml and consisting of various subfractions with specific properties [19]. Although the functions of the different HDL subpopulations remain largely unknown [20], the subclass of the large HDL is generally considered to be atheroprotective [21], while the intermediate and small HDL are considered more atherogenic [22], [23]. Few studies have so far assessed the effects of ezetimibe on HDL particle size and/or subclass distribution, with conflicting results [19], [24]–[27]. Furthermore, most of these trials included subjects with concomitant metabolic disorders such as obesity, hypercholesterolemia, diabetes, and the metabolic syndrome, and with a variety of co-medications having effects on lipoproteins. The purpose of the present study was to test the hypothesis whether simvastatin and/or ezetimibe modify HDL subclass distribution in healthy subjects with mild hypercholesterolemia.

## Subjects and Methods

The protocol for this trial and supporting CONSORT checklist are available as supporting information; see Checklist S1 and Protocol S1.

### Study design

HDL subfractions were analyzed from frozen samples of a single-center, randomized, parallel 3-group open-label study that investigated the effects of ezetimibe and simvastatin, alone or in combination, on lipid metabolism. The primary results of this randomized trial have been reported previously [14], [28], [29]. A total of 72 subjects were randomized to receive ezetimibe (10 mg/day), simvastatin (40 mg/day) or ezetimibe (10 mg/day) plus simvastatin (40 mg/day) for 2 weeks (n = 24 for each group). Ezetimibe and simvastatin were taken once a day in the evening. Blood was drawn before the initiation of treatment and at the end of the treatment period.

### Subjects

Inclusion criteria were age between 18 and 60 years, body mass index (BMI) between 18.5 and 30 kg/m^2^, fasting LDL-C concentrations <190 mg/dl, triglyceride (TG) concentrations <250 mg/dl and normal blood pressure (<140/90 mmHg). Subjects who had received lipid-lowering drugs within 12 weeks prior to study entry, those with a history of excessive alcohol intake, liver disease, renal dysfunction (estimated glomerular filtration rate <60 ml/min), coronary heart disease, diabetes mellitus or other endocrine disorders, eating disorders, history of recent substantial (>10%) weight change, history of obesity (BMI>35 kg/m^2^) or taking medications known to affect body weight or lipoprotein metabolism were excluded from the study.

The study protocol of the clinical trial was approved by the Ethics Committee of the University of Cologne, and all subjects gave written informed consent. The study has been conducted according to the principles expressed in the Declaration of Helsinki. All subjects completed the study. Body weight did not change in any treatment group. The subjects did not use any extra medications, had no illnesses and did not deviate from the study protocol. No serious side effects were reported.

### Biochemical analyses

Blood was drawn by venipuncture in the morning after a 12 h fast to obtain serum for analysis of lipids. Total cholesterol, LDL-C and HDL-C as well as TG were determined by enzymatic methods (CHOD-PAP and GPO-PAP; Roche Diagnostics, Mannheim, Germany) on the day of blood collection in the laboratories of the Cologne University Medical Center (inter-assay coefficient of variation for total cholesterol, LDL-C, HDL-C, and TG were 1.09, 2.79, 0.81, and 1.72%, respectively). Serum was obtained by centrifugation at 3000 rpm for 30 min at 4°C within 15 min after venipuncture and aliquots were stored immediately at −80°C for future analysis.

The Lipoprint system (Quantimetrix, Inc., Redondo Beach, CA) was used to measure HDL subfractions as previously described [30]. In short, the method uses polyacrylamide gel electrophoresis to separate HDL subfractions. The mobility of the HDL subfractions is identified using comparisons with LDL/VLDL as starting reference point and albumin (migrating the farthest) as leading reference point. Subfractions were quantitated using densitometric scanning. The relative area for each HDL subfraction band is determined and multiplied by the total HDL-C concentration in the sample to yield the amount of cholesterol for each band in mg/dl. Data for 10 subfractions are expressed in percent of the total. Fractions 1–3 are considered large HDL, fractions 4–7 intermediate and fractions 8–10 small HDL, respectively. This method has been validated against gradient gel electrophoresis and nuclear magnetic resonance and is the only FDA-approved diagnostic test for lipoprotein subclass testing in the United States [31]. Analyses were performed in the laboratory of M.R. and G.M. at the University of Palermo, Italy, in a blinded manner. Samples were shipped from Germany in dry ice. Previous studies have shown that freezing and thawing has no effect on the measurement of lipoprotein subfractions [32]. The coefficient of variation in repeated measurements was 1.2%.

Other biochemical analyses were performed as reported earlier [28], [29], [33]. Body composition was determined using bioelectrical impedance analysis.

### Statistical analysis

Descriptive data are presented as mean values ± SD unless otherwise stated. The primary outcome parameter was change in the proportion of the three main HDL subclasses. First, we dichotomized the subjects in the ones with increases or decreases in HDL subclasses, respectively. These outcomes were analyzed using contingency tables and calculating Pearson chi-square P-values.

The baseline proportions of large, intermediate and small HDL subclass compositions were then used in linear regression analyses to identify covariates that may influence therapy-induced changes. To assess the effects of treatment, we then constructed several multivariate models, always using HDL subclass composition after 2 weeks as the outcome and adjusting for baseline HDL subclass composition. In *model 1*, we investigated the effect of the three treatment arms only. In *model 2* we adjusted for several covariates that were either identified in univariate analyses or were expected to modulate the effects of treatment. *Model 3* was constructed to assess to what extent the effects of the 3 treatment arms were due to the respective changes in LDL-C from baseline. We therefore adjusted the final set of covariates used in *model 2* by percent change in LDL-C from baseline. We calculated the coefficients (and 95% confidence intervals) indicating the change in percentage HDL subclass composition from baseline. All models were stratified for large, intermediate and small HDL.

We performed several sensitivity analyses. First, we investigated change in HDL-C (as absolute amounts in mg/dl) as outcome parameter. Secondly, we combined intermediate and small HDL into one subclass. Thirdly, we used forward difference coding using the ezetimibe, simvastatin and combination groups as different levels of the LDL-lowering response.

Statistical analyses were conducted using Stata version 12 (StataCorp, College Station, TX). All reported P-values were calculated two-sided. Statistical significance was assumed at P-values<0.05.

## Results

**Figure 1:**
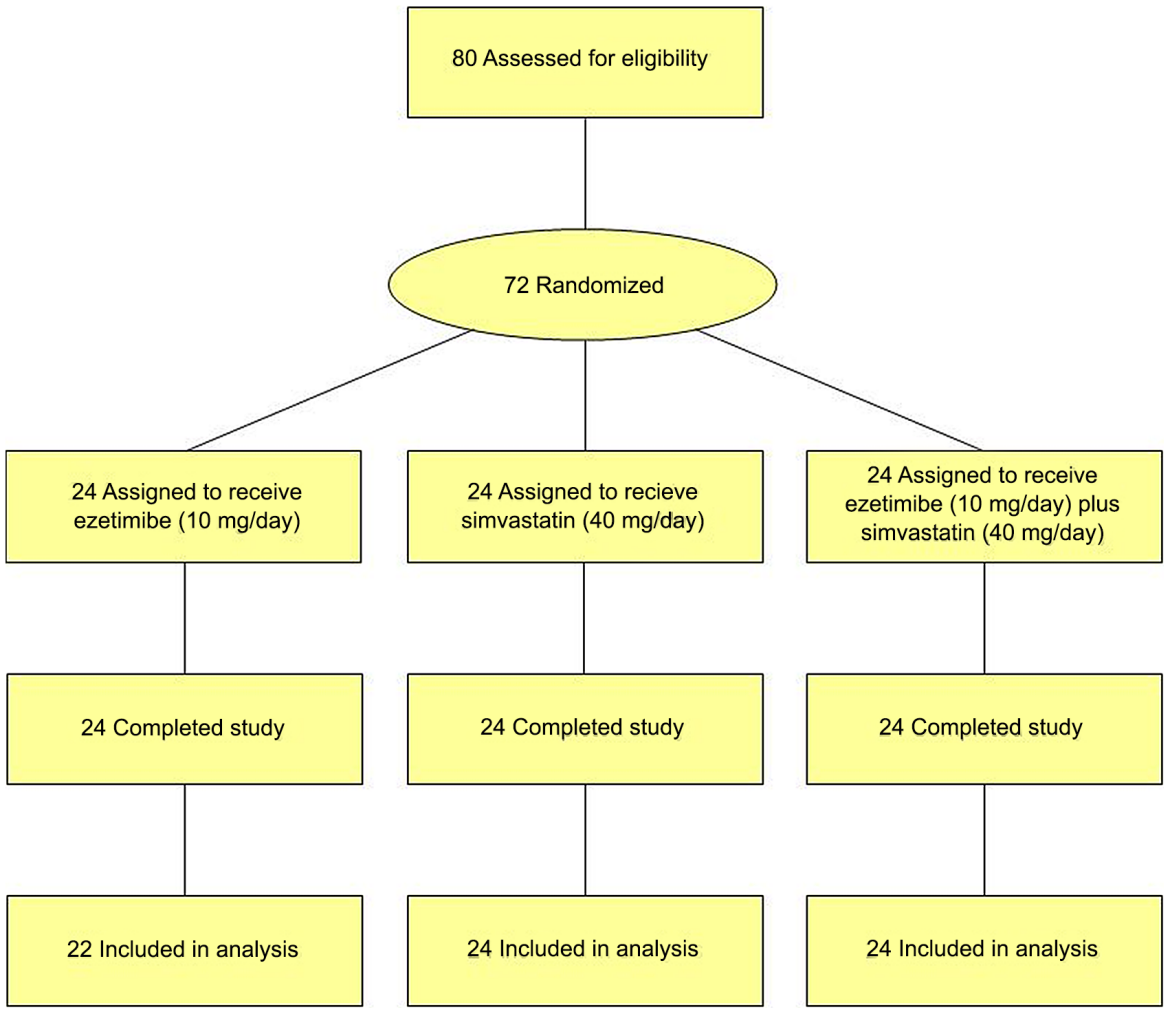
CONSORT flow diagram: Flow of participants through the trial.

The flow of participants through the trial is shown in **Figure 1**. All subjects completed the clinical study and their adherence was excellent, as based on pill counts (mean ± SD adherence, 99.1±3.7%). Baseline subject characteristics are shown in **Table 1**. All demographic and biochemical baseline parameters were similar between the groups. HDL subclass measurements at 2 weeks were not available in 2 of the 72 subjects.

**Table 1:** Demographic data and biochemical baseline characteristics of the study participants.

Parameter	Total cohort (n = 72)	Ezetimibe (n = 24)*	Simvastatin (n = 24)	Ezetimibe plus simvastatin (n = 24)
Age, yrs	32±9	29±7	32±9	34±11
Height, cm	181±7	181±7	182±6	181±7
Weight, kg	85±12	82±11	87±12	84±12
BMI, kg/m^2^	25.7±3.2	25.0±3.3	26.4±3.2	25.8±3.1
BIA body fat, %	21.4±5.7	20.6±5.4	22.5±5.7	21.1±6.2
BIA lean body mass, %	66.0±6.4	64.7±6.7	67.2±6.0	66.1±6.4
Fasting plasma glucose, mg/dl	88±8	87±6	86±7	89±2
Smoking status				
Current smoker, n (%)	21 (29)	7 (29)	8 (33)	6 (25)
Ex-smoker, n (%)	9 (12.5)	4 (17)	2 (8)	3 (13)
Never smoker, n (%)	42 (58.3)	13 (54)	14 (58)	15 (63)
*Serum lipoproteins*				
Total cholesterol, mg/dl	189±35	180±28	194±34	194±41
LDL cholesterol, mg/dl	111±30	105±23	113±30	116±35
HDL cholesterol, mg/dl	64±15	64±13	65±18	61±14
Triglycerides, mg/dl	95±43	78±32	101±45	106±48
Ratio total/HDL cholesterol	3.1±0.8	2.9±0.5	3.1±0.9	3.3±0.9
Ratio triglycerides/HDL cholesterol	1.6±1.0	1.3±0.6	1.7±1.1	1.9±1.0
*HDL subclasses*				
Large HDL, mg/dl (%)†	21.4±10.9 (32.7±9.9)	22.1±7.6 (35.3±9.0)*	21.9±14.9 (31.0±11.7)	20.2±8.9 (31.9±8.6)
Intermediate HDL, mg/dl (%)†	29.9±4.9 (48.3±5.7)	31.7±5.1 (51.2±4.7)*	29.7±5.0 (46.7±6.5)	28.4±4.3 (47.1±4.8)
Small HDL, mg/dl (%)†	11.8±5.3 (19.0±8.2)	8.3±4.5 (13.3±6.3)*	13.9±4.7 (22.2±8.2)	12.8±5.0 (21.0±7.2)

BMI, body mass index; BIA, bioelectrical impedance analysis; LDL, low-density lipoprotein; HDL, high-density lipoprotein.

Data are presented as mean ± SD or counts (percentages). There were no significant differences between the 3 treatment groups at baseline. Large HDL are composed of subclasses 1–3, intermediate HDL of subclasses 4–7 and small HDL of subclasses 8–10.

† percent of total HDL cholesterol.

*HDL composition data were not available in 2 subjects in the ezetimibe group.

At baseline, the subjects had mean proportions of large HDL of 32.7±9.9%, intermediate HDL of 48.3±5.7% and small HDL of 19.0±8.2%. There was significant inter-individual heterogeneity in HDL subclass patterns, as known and expected.

**Table 2** shows the correlations between baseline HDL subclasses and other parameters. These data were used to identify covariates for multivariate analyses.

**Table 2:** HDL subclasses baseline data and correlation with demographic and biochemical parameters.

	Large HDL			Intermediate HD			Small HDL		
	*Beta coefficient*	*R^2^*	*P-value*	*Beta coefficient*	*R^2^*	*P-value*	*Beta coefficient*	*R^2^*	*P-value*
*Demographic parameters*									
Age (years)	−0.31	0.07	0.02	−0.03	0.001	0.77	0.33	0.13	0.002
BMI (kg/m^2^)	−0.79	0.06	0.04	0.17	0.01	0.49	0.63	0.06	0.04
BIA body fat (%)	−0.68	0.14	0.001	0.37	0.10	0.01	0.31	0.05	0.07
BIA lean body mass (%)	−0.26	0.03	0.18	0.08	0.01	0.54	0.19	0.02	0.22
*Lipoproteins*									
Total cholesterol (mg/dl)	−0.04	0.02	0.28	−0.04	0.04	0.09	0.08	0.10	0.01
LDL cholesterol (mg/dl)	−0.13	0.13	0.002	0.01	0.003	0.66	0.11	0.16	<0.0001
HDL cholesterol (mg/dl)	0.40	0.34	<0.0001	−0.29	0.44	<0.0001	−0.12	0.04	0.08
Triglycerides (mg/dl)	−0.09	0.15	0.001	0.03	0.03	0.13	0.07	0.12	0.003
Ratio total/HDL cholesterol	−7.80	0.34	<0.0001	3.40	0.17	<0.0001	4.40	0.18	<0.0001
Ratio triglycerides/HDL cholesterol	−5.30	0.24	<0.0001	2.30	0.12	0.003	3.00	0.12	0.003
*Glucose metabolism*									
Glucose (mg/dl)	0.01	0.00	0.97	0.05	0.003	0.67	−0.05	0.002	0.71
Insulin (mU/l)	−0.30	0.06	0.04	0.21	0.07	0.02	0.09	0.01	0.46
HOMA	−1.36	0.06	0.05	1.00	0.08	0.02	0.37	0.01	0.52
*Adipokines*									
Leptin (µg/ml)	−0.82	0.05	0.07	0.39	0.03	0.16	0.43	0.02	0.22
Adiponectin (µg/ml)	0.52	0.07	0.02	−0.33	0.07	0.02	−0.20	0.02	0.28
Resistin (ng/ml)	0.38	0.04	0.10	−0.01	0.00	0.96	−0.38	0.06	0.04
*Other parameters*									
hsCRP (mg/l)	−0.31	0.08	0.02	0.08	0.01	0.35	0.24	0.07	0.03
PCSK9 (ng/ml)	−0.03	0.004	0.61	0.10	0.09	0.01	−0.07	0.03	0.18

The data are Pearson correlation coefficients. There were no significant correlations with (data not shown) estimated glomerular filtration rate, thyroid function tests, cholesterol synthesis and absorption markers, and smoking status.

HOMA, homeostasis model assessment; hsCRP, high-sensitivity C-reactive protein; PCSK9, proprotein convertase subtilisin/kexin type 9.

### Demographic parameters

Age and body mass index (BMI) were negatively correlated with large HDL and positively with small HDL. An increase of 10 years in age would lead to a decrease of 3.1 percentage points in large HDL. An increase of 5 kg/m^2^ in BMI would lead to a decrease of about 4 percentage points in large HDL. An even stronger negative correlation was observed between percent body fat and large HDL—an increase of 10 percentage points in body fat would be associated with a decrease of 6.8 percentage points in large HDL. Lean body mass was not significantly correlated with HDL subclasses.

### Lipoprotein concentrations

#### Large HDL

Significant positive correlations were observed between large HDL and HDL-C. Significant negative correlations were observed between large HDL and LDL-C, TG, the ratio total cholesterol/HDL-C and the ratio TG/HDL-C. For example, an increase of 10 mg/dl in HDL-C would be associated with an increase of 4 percentage points in large HDL. Vice versa, an increase of 50 mg/dl in TG would be associated with a decrease of 4.7 percentage points in large HDL.

#### Intermediate HDL

Significant positive correlations were observed between intermediate HDL and the ratios total cholesterol/HDL-C and TG/HDL-C. A significant negative correlation was found between intermediate HDL and HDL-C.

#### Small HDL

Significant positive correlations were observed between small HDL and total cholesterol, LDL-C, TG and the ratios of total cholesterol/HDL-C and TG/HDL-C.

### Glucose metabolism, adipokines, inflammation markers and other parameters

Insulin was negatively correlated with large HDL and positively with intermediate HDL. HOMA was negatively correlated with large HDL. Total adiponectin was positively correlated with large HDL and negatively with intermediate HDL. Resistin was negatively correlated with small HDL. High-sensitivity C-reactive protein was negatively correlated with large HDL and positively correlated with small HDL. Finally, proprotein convertase subtilisin/kexin type 9 (PCSK9) was positively correlated with intermediate HDL.

### Effects of drug treatment

As shown in **Table 3**, total cholesterol and LDL-C levels decreased significantly in all treatment groups (P<0.001 for all), while TG decreased only in the groups receiving simvastatin. HDL-C concentrations remained unchanged in all groups. The data on changes in lipoprotein concentrations have been published before [14], [28]. The changes in HDL subclasses are shown descriptively in **Table 3**.

**Table 3:** Plasma lipoprotein concentrations before and after treatment.

Parameter	Ezetimibe				Simvastatin				Ezetimibe plus simvastatin			
	*Before therapy*	*After therapy*	*Mean percent change*	*P-value*	*Before therapy*	*After therapy*	*Mean percent change*	*P-value*	*Before therapy*	*After therapy*	*Mean percent change*	*P-value*
Total cholesterol, mg/dl	180±28	159±23	−11.2±9.7	0.0002	194±34	145±24	−24.7±7.9	<0.0001	194±41	121±25	−36.9±8.1	<0.0001
LDL cholesterol, mg/dl	105±23	80±16	−22.1±10.2	<0.0001	113±30	67±22	−40.7±11.5	<0.0001	116±35	47±19	−59.6±9.7	<0.0001
HDL cholesterol, mg/dl	64±13	65±16	+1.7±11	0.35	65±18	65±16	+0.7±11.1	0.88	61±14	60±14	−1.5 ±8.5	0.23
Large HDL, mg/dl	22.1±7.6	21.2±8.1	−2.3±26.6		21.9±14.9	23.2±13.0	+15.9±32.0		20.2±8.9	21.4±8.1	+9.9±20.9	
Intermediate HDL, mg/dl	31.7±5.1	32.7±6.0	+3.4±13.1		29.7±5.0	29.7±4.4	+1.2±13.6		28.4±4.3	27.7±5.6	−2.4±11.3	
Small HDL, mg/dl	8.3±4.5	8.4±3.3	+13.6±48.2		13.9±4.7	12.0±4.3	−9.1±30.5		12.8±5.0	11.1±3.8	−8.8±26.1	
Triglycerides, mg/dl	75 (59, 96)	73 (57, 90)	+27±79	0.57	87 (69, 126)	74 (53, 97)	−11.8±39.9	0.04	100 (67, 139)	82 (63, 123)	−8.9±29.7	0.03

Data are presented as mean ± SD and in the case of triglycerides as medians (interquartile range). Each group comprised n = 24 subjects (HDL subclass data in the ezetimibe group were available in only n = 22 subjects).

**Figure 2:**
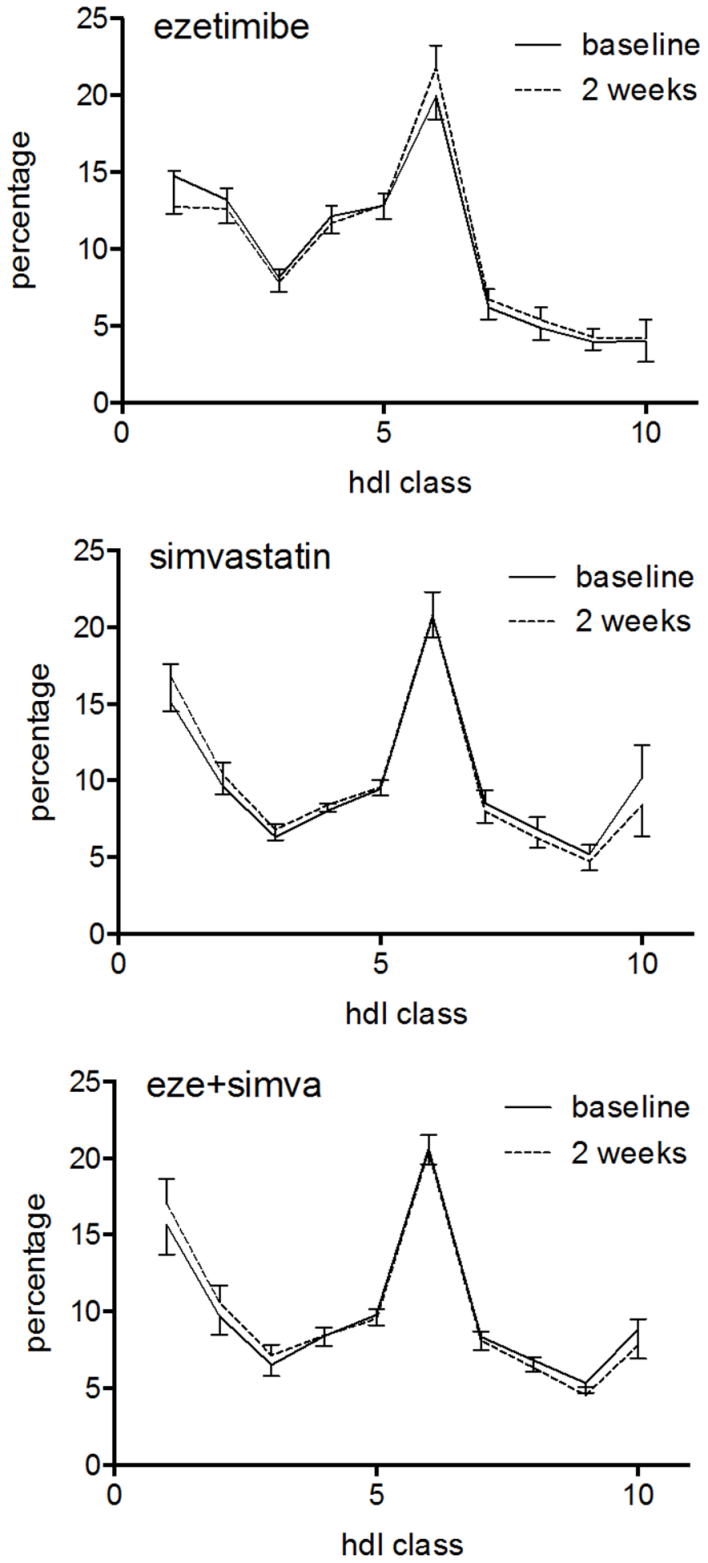
HDL subclass distribution before (solid line) and after 2 weeks of treatment (dotted lines). The data are means (95% confidence intervals) of the percentage of total HDL cholesterol. (A) ezetimibe, (B) simvastatin, (C) ezetimibe plus simvastatin.

**Figure 3:**
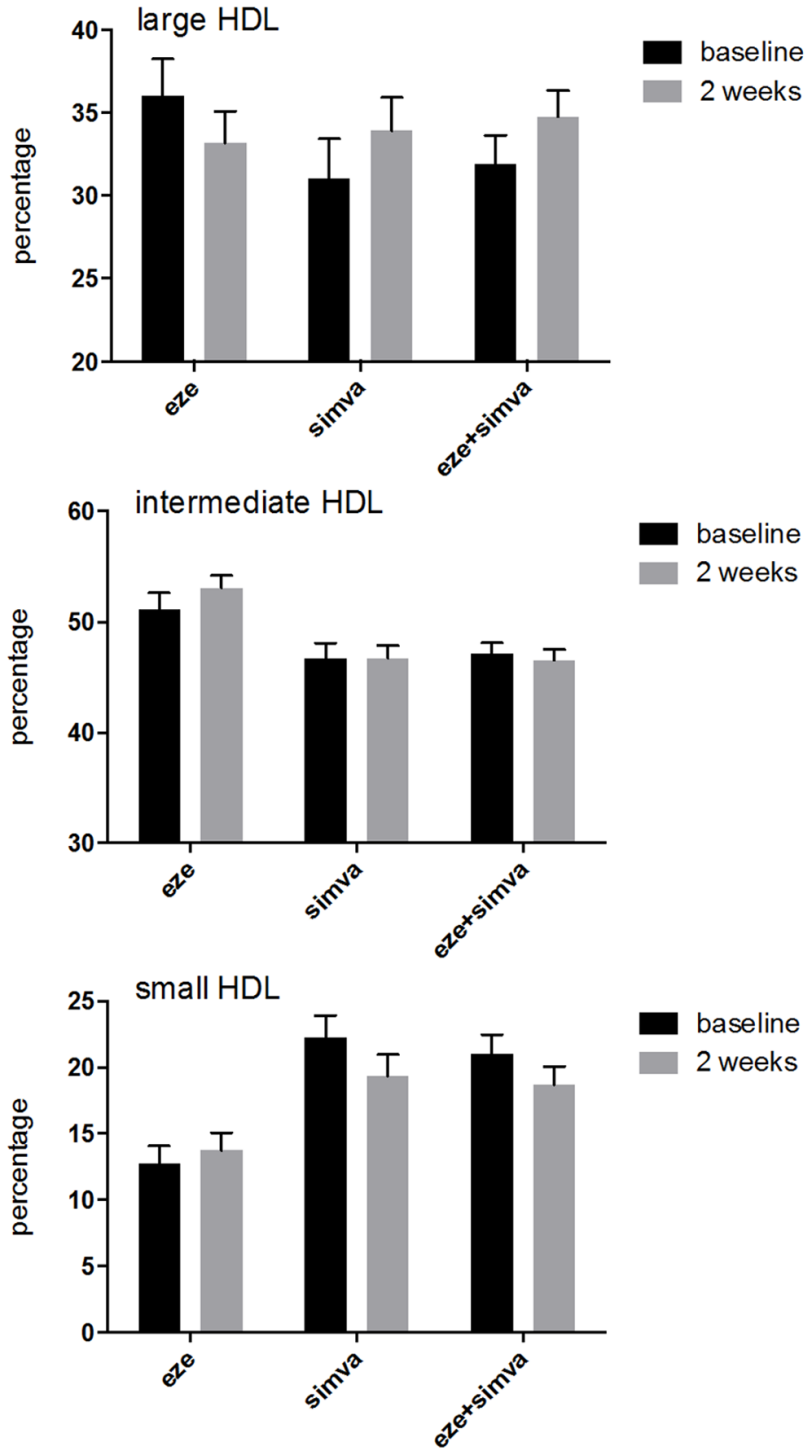
Mean (95% confidence intervals) proportions of HDL subclasses before and 2 weeks after treatment. (A) large HDL, (B) intermediate HDL, (C) small HDL.

**Figure 2** depicts the raw data of the 10 HDL subclasses, expressed as percentage of total HDL-C. The unadjusted data show that simvastatin treatment increased the proportion of large HDL and decreased small HDL, while ezetimibe seems to have opposite effects. **Figure 3** shows the changes summarized for large, intermediate and small HDL subclasses according to treatment. **Table 4** shows that the crude number of subjects with a decrease or increase in HDL subclasses was significantly different between the treatment groups. Simvastatin treatment increased large and decreased small HDL, while ezetimibe had the opposite effect.

**Table 4:** Effects of ezetimibe, simvastatin or the combination treatment on the number of subjects with an increase or decrease in HDL subfractions.

HDL subclass	Change	Treatment			P-value
		Ezetimibe	Simvastatin	Ezetimibe+simvastatin	
Large HDL	Increase	9	18	19	
	Decrease	13	6	5	P = 0.01
					
Intermediate HDL	Increase	13	12	11	
	Decrease	9	12	13	
					P = 0.66
Small HDL	Increase	13	6	9	
	Decrease	9	18	15	P = 0.06

The data indicate the number of subjects in the respective group. P-values were calculated using Pearson chi-square tests.

**Table 5** shows the results of multivariate regression modeling. In all models, HDL subclass composition after 2 weeks was the outcome parameter and all models were adjusted for the respective baseline HDL subclass composition. In *model 1* (basic model) we analyzed the main treatment effects and found significant effects on large and intermediate HDL. In comparison to ezetimibe, simvastatin or the combination significantly increased large HDL by 3.5 or 3.7 percentage points (P = 0.053 or 0.036, respectively) and significantly decreased intermediate HDL by 2.9 or 3.3 percentage points (P = 0.009 or 0.003, respectively). There was no significant effect observed on small HDL.

**Table 5:** Multivariate linear regression models.

	Beta coeff. (95% CI)		Beta coeff. (95% CI)			Beta coeff. (95% CI)	
	*Model 1*		*Model 2*			*Model 3*	
**Large HDL**	F(3, 66) = 29.8	R^2^ = 0.58 P<0.00001	F(6, 63) = 15.7	R^2^ = 0.60 P<0.00001		F(7, 62) = 13.6	R^2^ = 0.61 P<0.00001
Ezetimibe	reference		reference		Ezetimibe	−1.1 (−5.3 to 3.1)	P = 0.66
Simvastatin	3.5 (−0.1 to 7.0)	P = 0.05	3.8 (0.3 to 7.3)	P = 0.04	Simvastatin	1.4 (−4.8 to 7.6)	P = 0.66
Ezetimibe+simvastatin	3.7 (0.3 to 7.2)	P = 0.04	3.9 (0.4 to 7.4)	P = 0.03	Change in LDL cholesterol (per 20% decrease)	1.4 (−1.4 to 4.1)	P = 0.32
**Intermediate HDL**	F(3, 66) = 40.4	R^2^ = 0.65 P<0.00001	F(6, 63) = 20.3	R^2^ = 0.66 P<0.00001		F(7, 62) = 18.0	R^2^ = 0.67 P<0.00001
Ezetimibe	reference		reference		Ezetimibe	0.46 (−2.0 to 2.9)	P = 0.71
Simvastatin	−2.9 (−5.0 to −0.7)	P = 0.01	−3.0 (−5.3 to −0.6)	P = 0.01	Simvastatin	−1.4 (−5.2 to 2.4)	P = 0.46
Ezetimibe+simvastatin	−3.3 (−5.4 to −1.2)	P = 0.003	−3.5 (−5.9 to −1.8)	P = 0.004	Change in LDL cholesterol (per 20% decrease)	−1.1 (−2.7 to −0.5)	P = 0.16
**Small HDL**	F(3, 66) = 16.3	R^2^ = 0.43 P<0.00001	F(6, 63) = 9.7	R^2^ = 0.48 P<0.00001		F(7, 62) = 8.2	R^2^ = 0.48 P<0.00001
Ezetimibe	reference		reference		Ezetimibe	0.6 (−3.4 to 4.6)	P = 0.76
Simvastatin	0.3 (−3.5 to 4.0)	P = 0.89	−0.4 (−4.0 to 3.3)	P = 0.85	Simvastatin	0.5 (−5.6 to 6.6)	P = 0.86
Ezetimibe+simvastatin	0.3 (−3.3 to 3.9)	P = 0.86	−0.01 (−3.6 to 3.6)	P = 0.97	Change in LDL cholesterol (per 20% decrease)	−0.3 (−2.9 to 2.3)	P = 0.96

In *model 1*, only treatment group and the respective baseline HDL subclass composition was modelled. In *model 2*, BMI, baseline LDL cholesterol and the baseline ratio of triglycerides to HDL cholesterol were used as additional explanatory variables. In *model 3*, the change in LDL cholesterol was added as explanatory variable and the specific drug treatments were added as dummy variables.

*Note:* for ease of interpretation, coefficients for change in LDL cholesterol are given per decrease by 20% (corresponding to approximately the change in the ezetimibe group or one half of the change in the simvastatin group or one third of the change in the combination treatment group).

The ‘final’ *model 2* consisted only of BMI and the baseline lipoprotein concentrations (LDL cholesterol, HDL-C and TG) as covariates (in addition to baseline HDL subclass composition). TG and HDL-C were modeled as their ratio. No additional parameter described in **Table 2** contributed significantly to a further improvement in the model. BMI was a better predictor than percent body fat. Adjusting for these covariates, the coefficients obtained in *model 1* were further increased and their 95% confidence intervals decreased. The coefficients indicated that simvastatin or the combination therapy increased large HDL significantly by 3.8 or 3.9 percentage points (P = 0.035 or 0.031, respectively), while they decreased intermediate HDL by 3.0 or 3.5 percentage points (P = 0.013 or 0.004, respectively). The overall coefficients of determination in these models were 60 or 66%, respectively. Again, there were no significant effects on small HDL.

In the additional *model 3* we investigated to what extent the effects described by *model* 2 could be explained by the LDL-C-lowering effects of the treatments. Therefore, we adjusted for percent change in LDL-C from baseline. As indicated by the respective P-values, the effects of simvastatin and ezetimibe were not statistically significant any more in *model 3*. As indicated by the change in estimated coefficients, treatment with simvastatin would increase large HDL by 1.4 percentage points and decrease intermediate HDL by 1.4 percentage points. A decrease in LDL-C by 20% would thus decrease large HDL by 1.37 percentage points and increase intermediate HDL by 1.13 percentage points. The effects of ezetimibe tended to be decreasing large HDL (−1.1 percentage points) and increasing intermediate HDL (+0.5 percentage points). Again, no significant effects on small HDL were observed. The data of the adjusted analyses of *model 3* suggest that about one third to half of the effects of drug treatment on changes in large and intermediate HDL can be explained by the extent of LDL-C-lowering. The remaining half seems drug-associated and independent of lipid-lowering effects.

The results of the various sensitivity analyses (not shown in detail) did not alter the parameters obtained in multivariate analyses.

## Discussion

This *post hoc* analysis of a randomized study assessed the effects of 2 commonly used lipid-lowering drugs, simvastatin and ezetimibe, on HDL subclass distribution. The one essential finding of this study is that treatment with simvastatin alone or in combination with ezetimibe increased proportions of large HDL, thus resulting in a less atherogenic HDL subclass profile, since the proportion of large HDL particles seems to more accurately reflect the protective action of HDL than the levels of total HDL [34]. Ezetimibe alone did not show such effects. *Vice versa*, simvastatin decreased the proportion of intermediate HDL but ezetimibe did not.

The second important issue was to what extent the effects observed were caused by lowering LDL-C. As expected, the LDL-C-lowering effects in the 3 treatment arms were different in size (about 20% for ezetimibe alone, 40% for simvastatin alone and 60% for the combination). Adjusting for these changes, we found that up to one half of the effects of simvastatin could be explained by its LDL-C-lowering action. The reasons for the remaining half need to be clarified and are most likely due to the different mechanism of action of the two drugs.

Surprisingly, only a few studies investigated the effect of statins on HDL subclasses, and the results have not been concordant. Results of the clinical studies are nicely summarized in Harangi *et al.* [16]. For example, Cheung *et al.* [35] found that pravastatin 10 mg/day does not alter HDL subclasses in patients with mild to moderate primary hypercholesterolemia. On the other hand, Neuman *et al.* [36] had shown that simvastatin increased HDL_2_-C and decreased HDL_3_-C in spite of an increase in total HDL-C, suggesting that this effect indicates beneficial reverse cholesterol transport. However, Harangi *et al.* [37] showed that three months of treatment with atorvastatin 20 mg/day significantly increased the smaller HDL_3_ and decreased the larger HDL_2a_ and HDL_2b_ subclasses in patients with previously untreated hyperlipidemia type IIa/IIb. McKenney *et al.* [38] found that atorvastatin 10 mg/day in patients with atherogenic dyslipidemia slightly but significantly increased smaller HDL subclasses.

However, Kostapanos *et al.* [39] showed that rosuvastatin 10 and 20 mg per day increased large HDL particles in patients with primary hyperlipidemia. The CARDS investigators [40] examined the effect of 10 mg/day atorvastatin therapy on subclasses of HDL in a subset of 122 men and women with type 2 diabetes, modest dyslipidemia and previous myocardial infarction. Atorvastatin therapy was associated with a greater increase in large HDL than placebo and there was little change in small HDL so that average HDL particle size increased significantly with atorvastatin. Unfortunately, different statins have been administered at different doses in various patient populations and different assays were used, making the comparison of these results complicated and potentially misleading.

Regarding the effects of ezetimibe on HDL subclass distribution there is even less evidence available. Farnier *et al.* [41] found that the combination of ezetimibe (10 mg/day) and simvastatin (20 mg/day) did not change HDL subclasses compared to placebo in patients with mixed dyslipidemia. Kalogirou *et al.* [30] found in patients with primary dyslipidemia that treatment with ezetimibe significantly decreased small HDL. Since the methodology used was the same as in the current study, differences in population characteristics (e.g. age, BMI, presence of dyslipidemia) may explain the discordant results.

Nakou *et al.* [42], also using the Lipoprint system, found in overweight or obese subjects that, while orlistat increased large HDL and decreased intermediate and small HDL, ezetimibe monotherapy had no effects on large HDL but decreased intermediate and small HDL. Interestingly, the orlistat-associated increases in large HDL were not observed in the group treated with orlistat combined with ezetimibe.

Tomassini *et al.* [27] found that ezetimibe/simvastatin significantly increased small HDL in 1013 patients with type 2 diabetes and hypercholesterolemia compared to baseline while atorvastatin monotherapy had no effects.

### Clinical relevance

The clinical relevance of our findings remains to be established. Data regarding the predictive ability of HDL subclasses for CHD risk are inconclusive, although the concept that larger HDL particles may be associated with greater protection against atherosclerosis has been widely accepted (reviewed in [16]). However, Goliasch *et al.* [25] recently found no association between various HDL subclasses, also measured by the Lipoprint system, and the development of myocardial infarction at very young age (≤40 years of age). On the other hand, El Harchaoui *et al.* [24], utilizing the EPIC (European Prospective Investigation into Cancer and Nutrition)-Norfolk cohort, showed that both HDL size and HDL particle concentration were independently associated with other cardiovascular risk factors and with the risk for CAD. Moreover, in a much larger study (n = 5598 men and women without known CHD), the Multi-Ethnic Study of Atherosclerosis (MESA), Mackey *et al.* [12] demonstrated a consistent association between HDL-P and CHD risk. These findings disagreed with previous results of the Women's Health Study (WHS) [26]. In the trial involving 27,673 healthy women followed over an 11-year period, HDL-P was not associated with incident CVD.

In summary, the findings of the present study suggest that direct quantification of the HDL subclass composition may be useful to evaluate the role of established and novel HDL-directed therapies in the prevention of CVD and to identify differences between lipid-lowering drugs.

### Limitations and strengths of the study

A limitation of the study is the fact that the clinical relevance of our findings remains to be established. Strengths of the study include its randomized design and robust statistical methodology, the blinded measurements of HDL subclasses, and the use of a “drug-naïve” population, devoid of co-medications and co-morbidities, which could potentially alter lipid metabolism, and excellent treatment adherence. To our knowledge, the present study is the first one to examine whether ezetimibe modulates HDL size and subclass distribution in healthy individuals, a model which in a sense reflects ezetimibe's “true” effects on a normal metabolic background. Moreover, to the best of our knowledge this is also the first study that investigated effects of a statin on HDL subclasses in a healthy population with only mild hypercholesterolemia.

Treatment duration was relatively short, which does not exclude that the observed effects could be even more pronounced during long-term treatment, especially considering the different plasma residence times of HDL_2_ and HDL_3_. A two-week treatment duration was chosen for this study since the lipid-lowering effects of simvastatin reach maximum at day 14 and remain stable thereafter [43]. Regarding ezetimibe, Bays *et al.* [44] first showed that the maximum LDL-C-lowering effect is present after 2 weeks of treatment, after which it remains stable.

The methodological heterogeneity of HDL subclass assays is definitely one of the main limitations of using HDL composition to predict CVD risk. The laboratory assays of HDL subclasses include different methods based on variable HDL density, size, charge and composition [45]. In this work the Lipoprint system was used to analyze plasma HDL subclasses on polyacrylamide gel (PAG) [19]. This system has been widely used in the literature for this purpose [25], [30], [46]–[48].

Our results are in line with a very recent prospective cohort study showing that low baseline HDL-C levels were associated with increased CVD risk but in patients using intensive lipid-lowering statin therapy, HDL-C was not associated with recurrent CVD events, irrespective of LDL-C levels [49]. Beneficial statin-induced changes in HDL composition might be an explanation for these findings.

Interestingly, in the present rather small study in healthy (especially non-obese and non-diabetic) volunteers, the baseline associations with HDL subclass distribution pointed to the close interplay of HDL, TG, body composition, glucose metabolism, inflammation markers and adipokines. We observed for example that large HDL decreased with age, BMI, body fat, the ratio of total to HDL-C, insulin, HOMA, leptin and hsCRP.

In conclusion, our findings suggest that treatment with simvastatin alters HDL subclasses positively by increasing the proportion of large HDL. No such effect could be observed with ezetimibe. Since up to one half of the effect of simvastatin could be explained by its LDL-C-lowering action, additional ‘pleiotropic’ effects of statins may be implicated.

## Supporting Information

Checklist S1 **CONSORT Checklist.**
(DOC)

Protocol S1 **Trial Protocol.**
(PDF)
